# Towards Treatable Traits for Pulmonary Fibrosis

**DOI:** 10.3390/jpm12081275

**Published:** 2022-08-03

**Authors:** Thijs W. Hoffman, Jan C. Grutters

**Affiliations:** 1 Department of Pulmonology, St. Antonius Hospital, 3435 CM Nieuwegein, The Netherlands; t.hoffman@antoniusziekenhuis.nl; 2 Department of Pulmonology, Division of Heart and Lungs, University Medical Center Utrecht, Heidelberglaan 100, 3584 CX Utrecht, The Netherlands

**Keywords:** progressive pulmonary fibrosis, treatable traits, interstitial lung disease

## Abstract

Interstitial lung diseases (ILD) are a heterogeneous group of disorders, of which many have the potential to lead to progressive pulmonary fibrosis. A distinction is usually made between primarily inflammatory ILD and primarily fibrotic ILD. As recent studies show that anti-fibrotic drugs can be beneficial in patients with primarily inflammatory ILD that is characterized by progressive pulmonary fibrosis, treatment decisions have become more complicated. In this perspective, we propose that the ‘treatable trait’ concept, which is based on the recognition of relevant exposures, various treatable phenotypes (disease manifestations) or endotypes (shared molecular mechanisms) within a group of diseases, can be applied to progressive pulmonary fibrosis. These targets for medical intervention can be identified through validated biomarkers and are not necessarily related to specific diagnostic labels. Proposed treatable traits are: cigarette smoking, occupational, allergen or drug exposures, excessive (profibrotic) auto- or alloimmunity, progressive pulmonary fibrosis, pulmonary hypertension, obstructive sleep apnea, tuberculosis, exercise intolerance, exertional hypoxia, and anxiety and depression. There are also several potential traits that have not been associated with relevant outcomes or for which no effective treatment is available at present: air pollution, mechanical stress, viral infections, bacterial burden in the lungs, surfactant-related pulmonary fibrosis, telomere-related pulmonary fibrosis, the rs35705950 *MUC5B* promoter polymorphism, acute exacerbations, gastro-esophageal reflux, dyspnea, and nocturnal hypoxia. The ‘treatable traits’ concept can be applied in new clinical trials for patients with progressive pulmonary fibrosis and could be used for developing new treatment strategies.

## 1. Introduction

The current classification of human diseases, which is based on disease manifestations in specific organ systems with different physiological, anatomical, and histopathological correlates, does not seem to capture disease complexity for many types of diseases [1,2]. The definition of clinical phenotypes or endotypes, permitting the identification of distinct disease attributes that differentially respond to treatment in patients with the same type of disease, does not fully capture the biological complexities that lead to disease [2]. The ‘treatable traits’ concept has been proposed by Agusti for use in airway disease, and is distinct from endotyping or phenotyping [1,3]. Instead of requiring mutually exclusive phenotypes or endotypes, the ‘treatable traits’ concept can accommodate overlap between multiple traits in the same individual, regardless of a classifying diagnosis. Treatable traits should be clinically relevant, identifiable, and measurable through the use of validated biomarkers, and treatable [1,2]. This article explores how the ‘treatable traits’ concept can be applied to pulmonary fibrosis, a subgroup of interstitial lung diseases (ILD).

Histopathologically, ILD are characterized by either predominant inflammation or fibrosis, and, in the past, it has been proposed that they can be placed on an inflammation-fibrosis spectrum [4]. Diseases on one end of the spectrum, such as organizing pneumonia (OP), were considered to be solely inflammatory diseases, to be treated with immunosuppressive drugs. Diseases on the other end of the spectrum, such as IPF, were considered to be dominant fibrotic diseases, to be treated with anti-fibrotic drugs instead. Albeit helpful, the spectrum does not always capture the underlying ILD pathophysiology. Patients with the same type of ILD can show a wide range of disease behavior, varying from mild and stable to severe and rapidly progressive disease, including the potential for acute exacerbations of ILD. Patients with primarily inflammatory disease can also develop pulmonary fibrosis. In recent years there has been increased attention for the so-called progressive pulmonary fibrosis phenotype in patients with various types of ILD [5]. Several studies now suggest that anti-fibrotic drugs can be beneficial in patients with ILD that are usually thought to be on the inflammatory side of the spectrum such as ILD related to connective tissue disease (CTD-ILD), hypersensitivity pneumonitis (HP), sarcoidosis, and idiopathic non-specific interstitial pneumonia (NSIP) [6,7,8,9]. Apart from the previously mentioned diseases, other diseases that can manifest as progressive pulmonary fibrosis include pleuroparenchymal fibroelastosis, fibrosing organizing pneumonia, desquamative interstitial pneumonia, occupational interstitial lung disease such as asbestosis or pneumoconiosis, pulmonary Langerhans cell histiocytosis, pulmonary alveolar proteinosis, and unclassifiable interstitial lung disease [10]. A recent update of the Clinical Practice Guideline on diagnosis and treatment of IPF recommends anti-fibrotic treatment for patients with progressive pulmonary fibrosis in ILD other than IPF [10]. Consequently, developing personalized treatment strategies has become even more important.

It has been stated previously that treatable traits might at some point be identified in patients with progressive pulmonary fibrosis, but a concrete framework has not been developed [11]. In this perspective we propose that the ‘treatable traits’ concept can be applied to progressive pulmonary fibrosis and propose concrete treatable traits, as well as potential treatable traits. We think that the ‘treatable traits’ concept better reflects the complexity of pulmonary fibrosis pathophysiology and can be especially useful for the development of novel treatment strategies for patients with progressive pulmonary fibrosis.

## 2. Application of the ‘Treatable Traits’ Concept in Fibrotic ILD

We propose the following treatable traits for patients with progressive pulmonary fibrosis (Table 1): cigarette smoking, occupational, allergen or drug exposures, excessive (profibrotic) auto- or alloimmunity, progressive fibrosis, pulmonary hypertension, obstructive sleep apnea, tuberculosis, exercise intolerance, exertional hypoxia, and anxiety and depression. As has also been suggested for bronchiectasis [12], we have classified the traits into environmental factors, pulmonary disease, comorbidities and functional traits.

### 2.1. Environmental Traits

Cigarette smoking is a known risk factor for IPF, with evidence of a dose–response relationship [13]. Furthermore, there are several other ILD with a very strong etiological link to cigarette smoking: such as pulmonary Langerhans cell histiocytosis, respiratory bronchiolitis-associated ILD, and desquamative interstitial pneumonia. For patients with IPF, initial observations suggested that smoking is associated with a better prognosis, but this is likely related to the ‘healthy-smoker effect’, which represents less severe disease at diagnosis. When correcting for disease severity, survival actually seems to be worse in patients with IPF who are current smokers [14]. The detrimental effect of smoking on survival was recently demonstrated in patients with progressive pulmonary fibrosis due to non-IPF ILD [15]. There have been no randomized controlled trials investigating the effect of smoking cessation in patients with ILD, but smoking cessation alone has been associated with clear improvement in some patients with smoking-related ILD [13] and is likely beneficial for patients with all types of pulmonary fibrosis.

Numerous other exposures that can cause pulmonary fibrosis have been described, including radiation, asbestos, metal dust, wood dust, and various drugs [46,47,48,49,50,51]. Alternatively, many organic and inorganic compounds are also capable of inducing a lymphocyte-dependent immune response, which causes hypersensitivity pneumonitis. In these cases, it is not the offending antigen per se, but the local inflammatory response that can serve as a trigger for the fibrogenic response [52]. Continuous exposure to the offending agent has been associated with disease progression in HP, pneumoconiosis, and drug-induced ILD [17,18,19,20,21,22]. No randomized controlled trials of exposure avoidance have been performed, but removal of the offending exposure is very likely to be beneficial for all patients with pulmonary fibrosis related to specific exposures.

### 2.2. Pulmonary Traits

Persistent inflammation can be a feed-forward mechanism in pulmonary fibrosis. Several types of pulmonary fibrosis, such as CTD-ILD and HP are known to be caused by excessive auto- or alloinflammation [25,27]. Previous work has shown that alveolar macrophages from patients with several types of fibrotic lung disease show increased expression of the pro-Th2-inflammatory cytokine CCL 18, which seems to represent a feed-forward mechanism in the fibrotic process [53]. CCL18 expression has been associated with survival in patients with IPF and patients with CTD-ILD due to systemic sclerosis [54,55,56]. Gene expression studies performed on peripheral blood also support the role of inflammatory processes in progression of IPF [57,58,59,60], and in IPF lungs there is increased expression of genes related to inflammatory processes in fibrotic and non-fibrotic areas [61]. In a small cohort study, genes related to inflammatory processes were also upregulated in patients with fibrotic sarcoidosis and hypersensitivity pneumonitis [62]. Furthermore, it has been suggested that auto-inflammation might play a role in disease progression for patients with IPF, as increased numbers of circulating IgA auto-antibodies correlated with disease severity [63].

A caveat here is that inflammation is a broad concept, and inflammatory processes are a necessary part of the normal response to tissue damage and the normal fibrotic response [64]. Elevated markers of inflammation might simply represent more fibrosis, and do not necessarily imply a causative role for the inflammatory process in fibrosis progression. The presence of inflammatory cells in regions of tissue fibrosis can even be interpreted as their being part of an antifibrotic feedback loop [65]. The contribution of the immune system to the pathogenesis of pulmonary fibrosis is likely to be nuanced, as different immune cells can have either profibrotic or antifibrotic functions [66]. It will have to be determined what novel markers are able to distinguish a ‘normal’ inflammatory response from inflammation that is pathogenic and should be treated with anti-inflammatory drugs. At this time, anti-inflammatory treatment is known to be useful for patients with CTD-ILD, HP, and sarcoidosis, and can be considered for patients with idiopathic NSIP, but not in patients with IPF [25,27,67,68].

In recent years the progressive pulmonary fibrosis phenotype has been defined and is increasingly recognized. Independent of the underlying diagnosis, progressive pulmonary fibrosis despite optimal treatment is associated with a worse prognosis [28]. Progressive pulmonary fibrosis is defined as at least two out of three of: worsening symptoms, radiographic progression, and physiological progression [10]. Predictors of progressive pulmonary fibrosis include greater impairment in lung function, more extensive disease on CT-imaging, and the presence of honeycombing or a UIP pattern on imaging [5]. Several trials have now shown a benefit of treatment with the antifibrotic agents pirfenidone and nintedanib in patients with progressive pulmonary fibrosis [6,7,8,9], and this was consistent across subgroups of patients with different underlying diagnoses [69].

### 2.3. Comorbidities

Many different comorbidities can be found in patients with pulmonary fibrosis, but we have here only included comorbid conditions for which we think specific screening is warranted at this point. This means that a specific test is available to diagnose these conditions, and that effective treatment is available. Other comorbid conditions might be added in the future. Pulmonary hypertension is commonly observed in patients with pulmonary fibrosis, with the prevalence depending on the underlying diagnosis and the characteristics of the studied cohort [30]. For example, in patients with IPF who are listed for lung transplantation, the prevalence of pulmonary hypertension varies from 18–51% [30]. In patients with IPF, pulmonary hypertension is associated with shorter survival [30]. International treatment guidelines recommend treatment with pulmonary hypertension specific medication for patients with connective tissue disease and (possible) group 1 pulmonary hypertension (i.e., pulmonary hypertension due to inflammatory vasculopathy) [32]. The clinical practice guideline for IPF recommends against the use of pulmonary hypertension specific therapy for patients with IPF, and a recent trial failed to find a benefit of adding the pulmonary hypertension specific drug sildenafil to pirfenidone for patients with IPF [70,71]. A small randomized controlled trial suggests that inhaled nitric oxide might be beneficial in patients with pulmonary fibrosis complicated by pulmonary hypertension, but only used physical activity markers as outcome measures [72]. A recent large randomized controlled trial of inhaled treprostinill has showed that this leads to increased exercise tolerance in patients with group 3 pulmonary hypertension (i.e., pulmonary hypertension secondary to lung disease) [31], but it is not known if this improves survival.

Obstructive sleep apnea (OSA) has been observed in 32–85% of patients with IPF [33], but the prevalence in patients with other types of pulmonary fibrosis is not well-characterized. OSA in patients with IPF is associated with decreased survival [34], as well as the development of pulmonary hypertension [33]. Treatment of OSA with continuous positive airway pressure (CPAP) was associated with improved quality of life in patients with good treatment adherence, and might lead to better survival [35,36]. How screening for OSA can best be implemented for patients with pulmonary fibrosis remains to be determined.

Tuberculosis is a very common disease worldwide, although the prevalence in most Western countries is low [41]. It has been shown that patients with pneumoconiosis are at an especially high risk to develop active tuberculosis, and this is associated with worse survival [37,38]. In patients with IPF, the prevalence of active tuberculosis seems to be higher than in the general population, and this might also be associated with worse survival [39]. The presence of active tuberculosis may be suggested by radiographic abnormalities such as nodular abnormalities or consolidations, but can be difficult to distinguish from the fibrotic disease [40,73]. It remains to be determined if standard treatment regimens for tuberculosis should be extended for patients with pulmonary fibrosis, and if patients with pulmonary fibrosis would benefit from screening for and treatment of latent tuberculosis.

### 2.4. Functional Traits

Exercise intolerance has been associated with decreased quality of life in patients with ILD, and this can be improved by pulmonary rehabilitation [42]. Exertional hypoxia also leads to reduced exercise tolerance in patients with pulmonary fibrosis, and can be treated with supplemental oxygen during exercise [43]. It remains to be determined what the optimal pulmonary rehabilitation program is and what the best oxygen delivery system is. A randomized controlled trial of oxygen suppletion via a portable concentrator in patients with IPF is planned [74,75].

Clinically relevant anxiety and depression are found in 12 and 7% of patients with interstitial lung disease [44], and are associated with reduced quality of life [76]. A palliative care intervention that included a comprehensive assessment, care plan, and community case conference has been shown to improve anxiety and depression in patients with pulmonary fibrosis and caregivers [45]. Further development of care packages and trials of anti-anxiety medication in patients with pulmonary fibrosis are warranted.

### 2.5. Future Avenues for Detection and Treatment of Treatable Traits

As indicated in the table, several markers to distinguish these traits already exist. However, novel biomarkers will need to be developed to better identify the traits and associated response to treatment. In recent years, the term interstitial pneumonia with auto-immune features (IPAF) has been introduced in the context of research, and this might be a promising start to identify the inflammation trait, including distinguishing between normal or physiological inflammation and excessive or pathological inflammation [26]. Other promising avenues include the use of artificial intelligence for interpreting radiological imaging, as well as the study of new biomarkers in peripheral blood, bronchoalveolar lavage (BAL) fluid, or exhaled air [77,78,79,80,81,82,83,84,85,86,87]. Several new insights have been derived from single-cell transcriptomic analysis of fibrotic lung tissue, and this could lead to the identification and targeting of novel pathways involved in the fibrotic process [88]. Molecular classifiers derived from gene expression signatures in transbronchial biopsies can potentially be used to determine which non-IPF ILD patients will have progressive pulmonary fibrosis, without having to wait for the fibrosis to actually be progressive [89,90]. Therapeutic trials based on these markers will help guide treatment of patients with pulmonary fibrosis in the future.

### 2.6. Development of Novel Treatments within the Treatable Traits Framework

The treatable traits framework will also be useful for the development and evaluation of novel treatments for patients with progressive pulmonary fibrosis. Table 2 presents several possible traits that have been identified in patients with fibrotic ILD, but for which no clear association with relevant outcomes, clear method for detection, or proven treatment exists. This includes air pollution as an environmental trait, mechanical stress to the lungs, viral infections, bacterial burden in the lungs, surfactant-related pulmonary fibrosis, telomere-related pulmonary fibrosis, the rs35705950 *MUC5B* promoter polymorphism, and acute exacerbations of pulmonary fibrosis as pulmonary traits, gastro-esophageal reflux as a comorbidity, and dyspnea and nocturnal hypoxia as functional traits, although the latter could also be related to or grouped with OSA.

**Figure 1:**
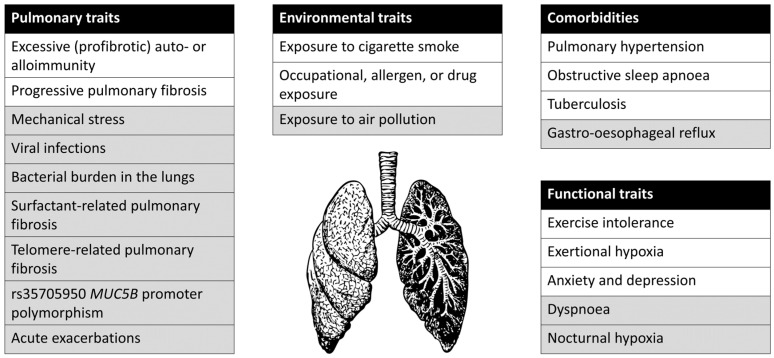
Proposed treatable traits for progressive pulmonary fibrosis, divided into pulmonary, environmental, functional, and comorbidity domains. Traits for which either a clear association with relevant outcomes, a clear method for measuring the trait, or a proven treatment is not available are marked in grey.

An overview of all proposed treatable traits, as well as potential treatable traits, is provided in Figure 1.

## 3. Discussion

Treatable traits are not yet a proven concept for management of progressive pulmonary fibrosis, and much will need to be done in order for this to happen. Some key points are presented in Box 1. There are also several other caveats to the treatable traits approach. First, not all traits presented here might be treatable in the foreseeable future, although fascinating new developments such as high-content screening using in-vitro cell-based assays might give unexpected acceleration [108]. Of course, the proposed set of traits is not fixed, and traits could be changed, added, or removed depending on future discoveries. Second, if many traits are implicated in the same patient, this could lead to a prohibitively complicated cocktail of therapies. Third, the traits can potentially be present to different degrees at different stages of the disease, and determining at which degree treatment for a given trait should be started could be difficult in practice. Finally, adopting a new classification will always be confusing initially, and should only be done when the clinical benefits are large enough to justify this [130]. Despite this, we think it is still worthwhile to further develop the treatable traits concept for pulmonary fibrosis. This approach is quite flexible, and the treatable traits model can be very useful to capture evolution of the disease process with time in an individual patient.

Box 1 **Developments necessary for the use of treatable traits for progressive pulmonary fibrosis in clinical practice.**

- Create larger patient cohorts through international collaboration, and study markers for disease behaviour in non-IPF fibrotic ILD
- Investigate in which patients immunosuppressive therapy is effective in addition to antifibrotic therapy
- Develop targeted therapies for fibrotic ILD patients with genetic abnormalities
- Investigate whether response to targeted therapies can be predicted by serum, BAL, or exhaled air biomarkers
- Investigate the value of molecular classifiers based on gene expression in transbronchial lung biopsies for identifying treatable traits
- Investigate the value of machine learning on radiological imaging for identifying treatable traits

In conclusion, there are many types of fibrotic lung disease, and there is large clinical variation in clinical presentation and disease course, even between patients with the same diagnosis under the current classification scheme. There are clear differences between the various diagnoses, but several processes seem to regularly play a role in patients with different types of progressive pulmonary fibrosis. These processes can be designated as treatable traits, which can be applied to all patients with progressive pulmonary fibrosis. Treatable traits are: cigarette smoking, occupational, allergen or drug exposures, excessive (profibrotic) auto- or alloimmunity, progressive fibrosis, pulmonary hypertension, obstructive sleep apnea, tuberculosis, exercise intolerance, exertional hypoxia, and anxiety and depression. There are also several potential traits that have not been associated with relevant outcomes or for which no effective treatment is available at present: air pollution, mechanical stress, viral infections, bacterial burden in the lungs, surfactant-related pulmonary fibrosis, telomere-related pulmonary fibrosis, the rs35705950 *MUC5B* promoter polymorphism, acute exacerbations, gastro-esophageal reflux, dyspnea, and nocturnal hypoxia.

This article tries to provide a comprehensive framework for viewing all types of progressive pulmonary fibrosis. There are several advantages of the treatable traits model: it is a flexible model, obviates the need to identify a single or several ultimate causes for pulmonary fibrosis, allows for the co-existence of multiple traits within the same patient, and allows for the existence of different traits at different times during the disease course. Furthermore, using treatable traits would make it easier to include patients with very rare or unclassifiable forms of pulmonary fibrosis under the present classification system in future clinical trials. In addition, the use of combination treatment for patients with progressive pulmonary fibrosis can be directed by the concept of treatable traits. This can pave the way for precision medicine in pulmonary fibrosis.

## Figures and Tables

**Table 1:** Treatable traits in patients with pulmonary fibrosis.

Trait	Association with Outcomes	How to Detect	How to Intervene	Future Avenues for Detection and Treatment
**Environmental**				
Cigarette smoking	Probably associated with worse survival in patients with IPF and other progressive fibrotic ILD [13,14,15], associated with disease progression in PLCH [16] and other smoking-related ILD	Smoking history	Smoking cessation	-
Occupational, allergen, or drug exposures	Ongoing exposure is probably related to survival in fibrotic HP [17,18,19,20] and to disease progression in pneumoconiosis [21] and drug-induced ILD [22]	Occupational, exposure, and drug history, serum IgG testing targeting potential antigens for HP [23]	Avoid relevant exposures	Development and validation of exposure questionnaire
**Pulmonary**				
Excessive (profibrotic) auto- or alloimmunity	CTD-ILD or HP has more favorable prognosis than IPF [24,25]	Established diagnosis of CTD, established diagnosis of HP, features suggestive of auto-immune disease, but no formal CTD diagnosis (features consistent with IPAF in clinical, serological or morphological domain) [26]	Immunosuppressive drugs [25,27]	Determine whether combination therapy of immunosuppressive drugs and antifibrotic treatment is warranted for certain patients, determine whether patients with certain features consistent with IPAF benefit from immunosuppressive treatment; investigate whether other circulating auto-antibodies can be used as a marker to give immunosuppressive therapy
Progressive fibrosis	Associated with increased mortality [28]	Two out of three of: worsening respiratory symptoms, physiological evidence of disease progression (absolute decline in FVC ≥ 5% of predicted within 1 year of follow up or absolute decline in DLCOc ≥ 10% of predicted within 1 year), or radiographical evidence of disease progression (increased extent or severity of traction bronchiectasis or bronchiolectasis, or new ground-glass opacity with traction bronchiectasis, or new fine reticulation, or increased extent or increased coarseness of reticular abnormality, or new or increased honeycombing, or increased lobar volume loss) [10].	Anti-fibrotic drugs [6,7,8,9], lung transplantation	Develop new radiological, histopathological, blood, BAL, or exhaled air biomarkers; develop novel strategies to replace fibrotic tissue with healthy tissue [29]
**Comorbidities**				
Pulmonary Hypertension	Associated with worse survival in patients with IPF [30]	Echocardiography, right heart catheterization	Consider inhaled treprostinil (associated with improved exercise capacity) [31]; consider PH-targeted therapy in patients with CTD-ILD and possible pulmonary arterial hypertension [32]; lung transplantation	Determine whether Treprostinil or inhaled nitric oxide leads to decreased mortality
Obstructive sleep apnea	Associated with decreased survival in patients with IPF [33,34]	Polysomnography	CPAP [35,36]	Determine how screening for obstructive sleep apnea can be implemented
Tuberculosis	Associated with decreased survival in patients with pneumoconiosis [37,38], might be associated with progression of IPF [39]	Can be suggested by CT-scan abnormalities [40], diagnosis by sputum or bronchial washing mycobacterial culture, molecular diagnostic tests [41]	Tuberculosis treatment depending on drug-sensitivity pattern	Determine if standard treatment regimens should be extended, determine if latent tuberculosis should be screened for
**Functional**				
Exercise intolerance	Reduced quality of life [42]	6-min walking test	Pulmonary rehabilitation [42]	Further development of specific pulmonary rehabilitation programs
Exertional hypoxia	Reduced exercise tolerance	Exercise testing (transcutaneous oxygen saturation ≤88% on 6-min walking test)	Ambulatory oxygen suppletion [43]	Optimize oxygen-delivery system
Anxiety and depression	Reduced quality of life	Hospital Anxiety and Depression Scale [44]	Palliative care intervention including assessment, care plan, and community case conference [45]	Further development of interventions to treat anxiety and depression

BAL = bronchoalveolar lavage; CPAP = continuous positive airway pressure; CTD-ILD = connective tissue disease-associated ILD; DLCOc = diffusion capacity of the lung for carbon monoxide, corrected for blood hemoglobin level; FVC = forced vital capacity; HP = hypersensitivity pneumonitis; ILD = interstitial lung disease; IPAF = interstitial pneumonia with autoimmune features; IPF = idiopathic pulmonary fibrosis; PH = pulmonary hypertension; PLCH = pulmonary Langerhans cell histiocytosis.

**Table 2:** Traits in patients with pulmonary fibrosis that are not (yet) treatable.

Trait	Association with Outcomes	How to Detect	Potential Avenues for Treatment
**Environmental**			
Air pollution	Associated with AE-IPF and progression of IPF [48,91,92,93,94,95]	Air quality monitoring, exposure history; no clear threshold for too much exposure	Possibly improve air quality, avoid exposure to bad quality air
**Pulmonary**			
Mechanical stress	Continuous mechanical stress is hypothesized to contribute to disease progression in patients with IPF [96,97], mechanical ventilation of patients with IPF is associated with high mortality [98,99]	Not clear	Avoid mechanical ventilation, possibly develop novel methods to decrease mechanical tension on alveoli
Viral infections	Potential role of human herpes viruses as a co-factor in initiation and progression of IPF [100,101]	Viral PCR on bronchoalveolar lavage fluid	Possibly antiviral treatment; no randomized controlled trials have been done yet
Bacterial burden in lungs	Bacterial burden in lower airways associates with disease progression in patients with IPF [51,102]	16s rRNA gene qPCR on bronchoalveolar lavage fluid	Possibly antibiotic treatment, vaccination; notably, treatment with cotrimoxazole or doxycycline had no effect on mortality or disease progression in patients with IPF [103,104,105]
Surfactant-related pulmonary fibrosis	Surfactant-related pulmonary fibrosis [106], higher risk of lung cancer in patients with *SFTPA2* gene mutations [107]	Mutations in *SFTPA1*, *SFTPA2*, *SFTPC*, *ABCA3*, *HPS*, *NKX2-1*	Development of novel treatments such as potentiators or gene-based therapy to correct surfactant processing [106], ABCA3 correction using cyclosporin A [108]
Telomere-related pulmonary fibrosis	Short leukocyte telomere length is associated with worse survival in patients with IPF or IPAF [109,110,111]; mutations in telomere-related genes are associated with a worse prognosis in patients with pulmonary fibrosis [112]	Telomere gene mutations, very short leukocyte telomere length [113]	Investigate anti-aging and telomere lengthening treatments such as dasatinib/quercetin [114], danazol [115], telomerase transfection [116]
rs35705950 *MUC5B* promoter polymorphism	Possibly associated with better survival in patients with IPF [117,118] and NSIP [119]	Genotyping of rs35705950 *MUC5B* promoter polymorphism	Investigate whether treatment with N-acetylcysteine [120], P-2 [119,121] or other mucolytics is effective
Acute exacerbation	Very poor prognosis in various types of ILD [122]		Further investigation of factors predicting acute exacerbation of pulmonary fibrosis such as lymphocytosis in bronchoalveolar lavage fluid [123]; investigate novel treatments
**Comorbidities**			
Gastro-esophageal reflux	Possibly associated with acute exacerbations or disease progression in patients with IPF [124]	24-h pH monitoring, patient history	Antacid therapy might be helpful and was conditionally recommended in IPF treatment guidelines [125], however there are increasing signals that this is not effective [126], and it is no longer recommended in updated guidelines [10]; laparoscopic fundoplication was not found to affect disease progression or mortality in patients with IPF in a small randomized controlled trial [127]
**Functional**			
Dyspnea	Reduced quality of life [128]	Clinical history	Investigate whether benzodiazepines and/or opioids can safely be used for symptom relief
Nocturnal hypoxia	Early mortality [129]	Polysomnography	Investigate efficacy of nocturnal oxygen suppletion

AE-IPF = acute exacerbation of IPF; ILD = interstitial lung disease; IPAF = interstitial pneumonia with autoimmune features; IPF = idiopathic pulmonary fibrosis; PCR = polymerase chain reaction.
